# Evaluation of modeling strategies for assessing self‐thinning behavior and carrying capacity

**DOI:** 10.1002/ece3.4525

**Published:** 2018-11-11

**Authors:** Christian Salas‐Eljatib, Aaron R. Weiskittel

**Affiliations:** ^1^ Centro de Modelación y Monitoreo de Ecosistemas Facultad de Ciencias Universidad Mayor Santiago Chile; ^2^ Laboratorio de Biometría Departamento de Ciencias Forestales Universidad de La Frontera Temuco Chile; ^3^ School of Forest Resources University of Maine Orono ME

**Keywords:** competition, density trajectories, density‐dependent, mixed‐effects models, mortality, negative binomial model, quantile regression

## Abstract

Self‐thinning and site maximum carrying capacity are key concepts for understanding and predicting ecosystem dynamics as they represent the outcome of several fundamental ecological processes (e.g., mortality and growth). Relationships are often derived using alternative modeling strategies, depending on the statistical approach, model formulation, and underlying data with unclear implications of these various assumptions. In this analysis, the influence of contrasting modeling strategies for estimating the self‐thinning relationship and maximum carrying capacity in long‐term, permanent plot data (*n *=* *130) from the mixed *Nothofagus* forests in southern Chile was assessed and compared. Seven contrasting modeling strategies were used including ordinary least squares, quantile, and nonlinear regression that were formulated based on static (no remeasurements) or dynamic data (with remeasurements). Statistically distinct differences among these seven approaches were identified with mean maximum carrying capacity ranging from 1,050 to 1,912 stems/ha depending on the approach. The population‐level static approach based on quantile regression produced an estimate closest to the overall mean with site‐level carrying capacity depending on tree species diversity and climate. *Synthesis and applications*. Overall, the findings highlight strong variability within and between contrasting methods of determining self‐thinning and site maximum carry capacity, which may influence ecological inferences.

## 1 INTRODUCTION

Size–density relationships are essential for understanding and predicting core ecological processes. These relationships highlight how the number of individuals in a population decreases with the progression of time, or more specifically as the individuals increase their average size. Therefore, a size–density relationship is a fundamental result of highly dynamic competition and mortality processes. This concept is an essential aspect of many ecological disciplines including forest (Jack & Long, 1996), wildlife (Jonsson, 2017), and fisheries ecology (Elliott, 1993). Self‐thinning results from a frontier relationship between stand density and tree size (Bi et al., 2000). That is, self‐thinning occurs when the population density reaches the maximum possible for a given average individual size, and so any increase in average size causes a decline in stand density. The self‐thinning phenomenon is one of the few fundamental rules throughout ecology and has led to several important concepts such as the ‐3/2 law for plants (Reineke, 1933; Yoda et al., 1963), ‐4/3 power rule for animals (Begon et al., 1986), and maximum carrying capacity (Enquist et al., 1998). Although the ‐3/2 power rule has received some criticism (Lonsdale, 1990; Weller, 1987), its use for ecological interpretation of population dynamics is widely utilized and accepted by ecologists.

The carrying capacity of a population can be expressed in the same way as the asymptote of a logistic equation in an ecological model (Gore & Paranjpe, 2001; Gotelli, 2001; Pielou, 1977), as the maximum density for a given average individual size. This value is generally termed the maximum stand density index (*SDI*_*max*_), and it is used for a range of purposes (Avery & Burkhart, 2002; van Laar & Akça, 2007). The maximum carrying capacity of a forest has been recently shown to vary depending upon species composition (Binkley, 1984; Puettmann et al., 1992; Stout & Nyland, 1986), species functional traits (Ducey et al., 2017), and climate factors (Weiskittel et al., 2009). In addition, its potential uses for multicohort and structurally complex forests has been assessed, and general findings suggest a similar usefulness as for more simpler forests (Ducey & Knapp, 2010; Sterba & Monserud, 1993). Construction of maximum size–density relationships provides a basis for quantifying the ecological concepts of self‐thinning and carrying capacity. However, defining this frontier relationship is difficult and, consequently, it has been derived using a variety of different approaches that depend upon the statistical approach, model formulation being used, and the actual data source.

Several statistical models have been proposed for fitting self‐thinning relationships. The most common approach is to fit a base model, for example, the logarithmic of Reineke (1933), using ordinary least squares (OLS) and then shift the intercept upwards by computing the upper level of the 95% confidence interval for that estimated parameter. Nevertheless, this approach does not fully account for the structure of the data, and more suitable statistical models for building self‐thinning lines have been devised. Zhang et al., (2005) compared different alternatives for estimating the self‐thinning boundary line including the following: OLS, corrected OLS, deterministic frontier function (DFF), stochastic frontier function (SFF) or stochastic frontier regression (SFR), and quantile regression (QR). Their results favored SFR, although QR performed nearly as well. VanderSchaaf and Burkhart (2007) elaborated further on the biological implications of the maximum size—density relationships, and compared OLS, a first‐difference model, and linear mixed‐effects (LME) models for fitting this relationship. They found that the LME was a better alternative for estimating the slope of the Reineke's model in specific situations without accounting for self‐thinning patterns of individual stands. Remeasured or dynamic data can be used to approximate an instantaneous growth rate. Vanclay and Sands (2009) applied this concept, for analyzing the self‐thinning frontier. In contrast, Weiskittel et al., (2009) used static data and SFR for estimating the self‐thinning boundary line in different forest types in the Pacific Northwest, USA. They also found that site productivity and the proportion of basal area of the primary species being modeled were important predictor variables for the size–density relationship. Recently, Andrews et al., (2018) used QR with mixed‐effects to determine carrying capacity for several common species in the Acadian Region of North America and found the method to provide robust site‐level estimates that were influenced by a variety of factors including species functional traits and climate.

Similar to alternative statistical approaches, different data sources have been used for developing self‐thinning relationships. The most common type of data for fitting size–density models is from static measurements that lack repeat observations. Static data have been primarily used as they are easy to collect and can be quickly assessed over a wide range of conditions, whereas their primary disadvantage for constructing a self‐thinning relationship is that either a full range of conditions must be measured, particularly high‐density sites, or an appropriate statistical model for estimating it be used. Dynamic data contain site‐level attributes that have been remeasured through time. This source of data has been proposed by García (2009) for developing self‐thinning lines, based upon differential equations. Recently, Trouve et al., (2017) followed García's (2009) approach in single‐species forests in Australia and found that the dynamic approach performed similar to a static one, which was similar to the findings of Kweon and Comeau (2017). Smith and Hann (1984) also suggested the use of dynamic data for developing maximum size–density models, which was later modified by Hann et al., (2003) for using the first site‐level measurement along with future observations to determine a site's trajectory in self‐thinning space. The third type of data proposed for self‐thinning studies are individual tree‐level observations. For example, Ducey and Knapp (2010) proposed this approach as a way for estimating the maximum size–density relationships in mixed species and structurally complex forests. The method was later generalized further by Ducey et al., (2017) for incorporating climate and species functional traits.

A variety of core questions remain on the various estimation strategies for modeling self‐thinning relations and site‐level maximum carrying capacity. The vast majority of research on this topic had been conducted for single‐species stands, but relatively limited research has been taken for mixed‐species forests, particularly species rich and productive temperate rainforests. In addition, the dependency of site‐level carrying capacity on climate conditions and other environmental factors has rarely been taken into account. As indicated by Weiskittel et al., (2009), most studies have used subjective or significantly limited statistical techniques for fitting the self‐thinning line. Furthermore, most studies have used static data, but rather few have explored and compared the use of dynamic data for estimating maximum size–density models. Finally, most studies have ignored the hierarchical structure in both static and dynamic data, which may have biased findings and limit general inferences to population‐ rather than site‐level trends. Therefore, we aimed to: (a) develop alternative strategies for constructing the maximum size–density relationship that explicitly account for hierarchical data; (b) compare implied estimates of site‐level maximum carrying capacity; and (c) relate observed carrying capacity to various site‐level and environmental variables.

## 2 METHODS

### 2.1 Data

Our study area covers the secondary *Nothofagus* forests in the southcentral part of Chile (37°–41°S.) Specifically, we focus on the *N. obliqua*,* N. alpina*, and *N. dombeyi* forest type (Donoso, 1995), which covers around 600,000 ha. These forests are part of the temperate rainforests of Chile, which represents the second largest remaining area of this type in the world (Donoso, 1995; Wilcox, 1995) and are internationally recognized for their ecological importance (Davis, Heywood et al., 1994; Olson & Dinerstein, 1998; Stattersfield, 1998). As highlighted by Salas et al., (2016), these three species are the most important for commercial and cultural purposes, which are usually located on the most productive sites in the Central Depression and foothills of the Andes.

**Figure 1:**
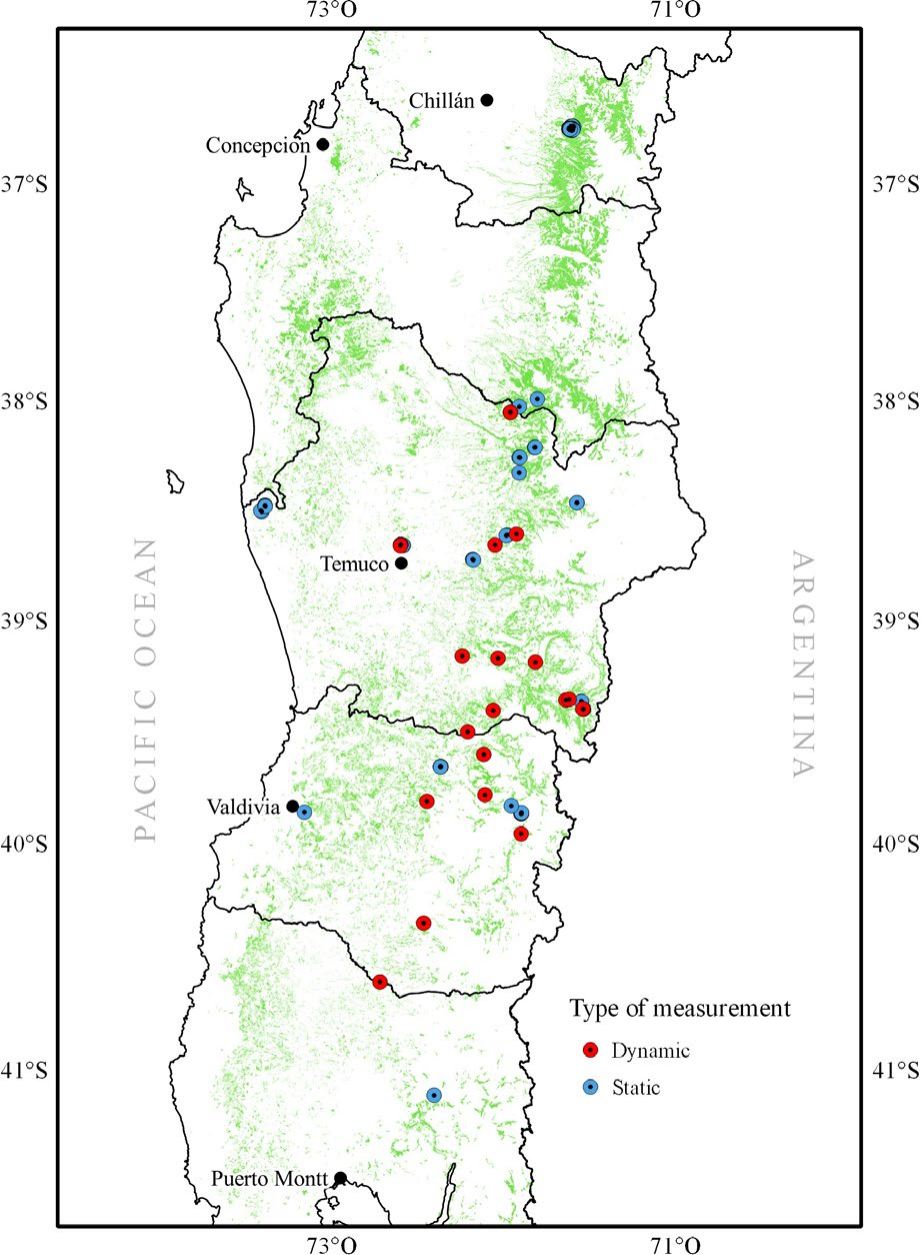
Permanent sample plots distribution (dots) in secondary *Nothofagus* forests (green) in south‐central Chile. The plots with remeasurements on time are mark as “dynamic” or “static” otherwise

We use data from permanent sample plots established throughout southcentral Chile where the roble‐raulí‐coigue forest type grows (Figure 1). The plots areas ranged from 500 to 10,000 m^2^ and were based on conventional tree‐level measurements of trees larger than 1.3 m in height with a diameter at breast‐height (*d*) greater or equal to 5 cm. We computed stand variables at the plot‐level (e.g., density *N* and diameter of the mean basal area tree *d*_*g*_). Plots remeasured at least once were considered as “dynamic data,” while others provided “static data.”

**Figure 2:**
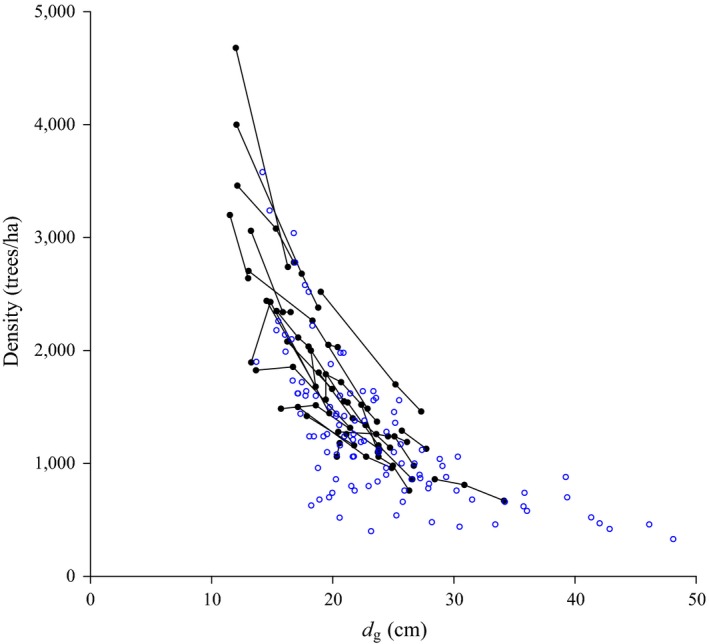
Population density versus average individual size (*dg* is the quadratic mean diameter) for permanent sample plots of *Nothofagus* forests. Dots joined by lines correspond to remeasured plots (i.e., dynamic), and single dots represents plots without remeasurements (i.e., static)

The dynamic plots are clearly shown as a time series, meanwhile the static plots are only shown as single dots. Figure 2 shows the relationship between density and quadratic mean diameter (i.e., a plot‐level averaged tree diameter), highlighting the progressive decrease in individuals in the population as they increase their size. The descriptive statistics for the primary site‐level variables by type of data are summarized in Table 1. We have a total of 130 plots, and because some of them have been remeasured, the total number of observations is 178. Note that from the available plots, 26 of them have more than one measurement.

**Table 1:** Descriptive statistics for the primary forest variables segregated by measurement type of plots: static (i.e., no remeasurements) and dynamic (i.e., at least one remeasurement)

Statistics	Type of measurement							
	Static (*n* = 104)				Dynamic (*n* = 74)			
	*N* (trees/ha)	*d*_*g*_ (cm)	*G* (m^2^/ha)	PBA (%)	*N* (trees/ha)	*d*_*g*_ (cm)	*G* (m^2^/ha)	PBA (%)
Minimum	330	13.7	16.4	48.0	670	11.5	26.2	39.7
Average	1,270	23.7	49.5	77.3	1,770	20.2	50.7	82.5
Median	1,160	21.8	47.5	82.5	1,530	20.1	50.3	86.6
Maximum	3,580	48.1	106.4	100.0	4,680	34.1	85.5	100.0
CV (%)	50	28.6	32.8	25.9	44	23.8	23.0	17.5

Note

The variables are population density (*N*; trees/ha), quadratic mean diameter ($d_{g}$; cm), basal area (G; m^2^/ha), and percentage of basal area of *Nothofagus* species (PBA; %).

Besides variables representing forest features, we also obtained bioclimatic variables for each plot location from WorldClim (http://www.worldclim.org) surfaces (Hijmans et al., 2005). These 19 bioclimatic variables represent annual trends (e.g., mean annual temperature, annual precipitation), seasonality (e.g., annual range in temperature and precipitation), and extreme or limiting environmental factors (e.g., temperature of the coldest and warmest month, and precipitation of the wet and dry quarters). Note that these bioclimatic variables were long‐term averages (Hijmans et al., 2005). In addition, topographic features such as elevation, aspect, and slope were also evaluated.

### 2.2 Modeling strategies

We evaluated different alternatives for constructing and interpreting self‐thinning relationships, which were a combination of data source, model form, and statistical method. The data sources are defined by the type of measurement (i.e., static or dynamic) and the level of information (i.e., site‐ or individual‐level). The different modeling strategies are summarized in Table 2 and explained further in the following paragraphs.

**Table 2:** Modeling strategies evaluated in this analysis. They were a combination between the data source, model form, and the statistical model being used

Modeling approach acronym	Data source		Statistical model	Equation number
	Type of measurement	Level		
S‐P‐LME	Static	Plot	LME	(2)
S‐P‐LQMM		Plot	QR	(3)
S‐P‐SFMM		Plot	SF	(4)
S‐T‐LME		Tree	Ducey	(5), (6)
D‐P‐LME	Dynamic	Plot	Density	(10)
D‐P‐NBME		Plot	Mortality	(13)
D‐P‐NLME		Plot	1st measu.	(14)

1. *Static, site‐level data, and linear mixed‐effects fit (S‐P‐LME)*: We used the Reineke's formulation (Reineke, 1933), as the base model for representing the relationship between stem density—average tree size, as follows
  (1) $$\ln N_{\mathrm{ij}}=\beta_{0}+\beta_{1}\ln d_{g_{\mathrm{ij}}}+e_{\mathrm{ij}},$$
  where: $N_{\mathrm{ij}}$ and $d_{g_{\mathrm{ij}}}$ the tree density and the quadratic mean diameter for the j‐th measurement at the i‐th plot, respectively; while $e_{\mathrm{ij}}$ is a random error following a Gaussian distribution having an expected value of 0 and variance $\sigma_{e_{\mathrm{ij}}}^{2}$. VanderSchaaf and Burkhart (2007) proposed a method for estimating maximum size—density lines based on a mixed‐effects model by adding random effects into both parameters of the Reineke's model, as follows,
  (2) $$\ln N_{\mathrm{ij}}=\left(\beta_{0}+u_{0i}\right)+\left(\beta_{1}+u_{1i}\right)\ln d_{g_{\mathrm{ij}}}+e_{\mathrm{ij}},$$
  where $u_{0i}$ and $u_{1i}$ are plot‐specific random effects to be predicted and assumed to follow a Gaussian distribution having an expected value of 0 and variance $\sigma_{0}^{2}$ and $\sigma_{1}^{2}$, respectively. We fit this model (Equation (2)) using the nlme package (Pinheiro & Bates, 2000) of R Core Team (2017). In order to estimate the self‐thinning line, we computed the upper level of the 95% confidence interval for the estimated intercept‐coefficient of the model, by adding the estimated parameter to its respective standard error multiplied by 1.96 (i.e., the quantile from the *t*‐distribution for α=0.05).
2. *Static, site‐level data, and quantile regression fit* (S‐P‐LQMM): Both OLS and LME aim at estimating population‐averaged parameters and therefore do not directly model the frontier relationship that are of interest in self‐thinning estimation. A regression equation that models the median instead of the expected value, such as a conditional‐mean regression would be a more suitable alternative for a frontier relationship. Koenker and Bassett (1978) proposed a more general model, the quantile regression model (QR). The QR corresponding to a mean conditional model can be expressed as the *p*th conditional quantile given $x_{i}$ as:
  (3) $$Q^{\left(p\right)}\left[y_{\mathrm{ij}}|x_{\mathrm{ij}}\right]=\beta_{0}^{\left(p\right)}+\beta_{1}^{\left(p\right)}x_{\mathrm{ij}}+Q^{\left(p\right)}\left(\epsilon_{\mathrm{ij}}\right),$$
  where $Q^{\left(p\right)}\left[y_{\mathrm{ij}}|x_{\mathrm{ij}}\right]$ is the *p*‐th quantile for $y_{\mathrm{ij}}$ being determined by the quantile‐specific parameters $\beta_{0}^{\left(p\right)}$ and $\beta_{1}^{\left(p\right)}$, and the value of $x_{i}$, where $y_{\mathrm{ij}}=\ln N_{\mathrm{ij}}$ and $x_{\mathrm{ij}}=\ln d_{g_{\mathrm{ij}}}$ as in the Reineke's model. We fit the quantile regression model (3) in a mixed‐effects framework by adding random effects (plot‐specific) to the intercept of the model, therefore fitting a linear quantile regression mixed‐effects model (LQMM). We tested different quantile values as recommended by Ducey and Knapp (2010). We choose the quantile that gave us the lowest variance estimates for the estimated parameters. According to our analyses, the 0.95 quantile was selected. This quantile mixed‐effect model was fitted by assuming a log‐likelihood expression based on an asymmetric Laplace density function, as suggested by Geraci and Bottai (2007), and using the lqmm package (Geraci, 2014) implemented in R. Because the quantile regression model was fitted in a mixed‐effect framework, we should not simply use the fixed‐effect estimated quantile‐intercept parameter as the corresponding parameter for constructing the self‐thinning line. This would be misleading in the sense that it does not fully represent the quantile‐parameter itself, but an average of it. Therefore, as in the above strategy, we computed the upper level of the 95% confidence interval of the intercept parameter for the LQMM model.
3. *Static, site‐level data, and stochastic frontier regression fit (S‐P‐SFMM)*: Stochastic frontier regression (SFR, Aigner et al., 1997) is an econometrics model that is often used to determine the technical efficiency of business firms but has been used in the past for self‐thinning analyses (Bi et al., 2000; Weiskittel et al., 2009; Zhang et al., 2005). We express a SFR model as the following matrix model
  (4) $$Y=X\beta +\left(V-U\right),$$
  where: the vector Y contains all the $\ln N_{\mathrm{ij}}$; X is the design matrix having the observations of $\ln d_{g_{\mathrm{ij}}}$; β is a vector of coefficients; the vector V is a random variable representing a portion of the model error, where $V_{\mathrm{ij}}∼N\left(0,\sigma_{V}^{2}\right)$; the vector U is a positive random variable representing the other portion of the model error following a half‐Gaussian with $U_{\mathrm{ij}}∼\left|N(0,\sigma_{U}^{2})\right|$. In this analysis, a true random effects SFR model of Greene (2005) was used to account for the data hierarchy. The SFR model (4) in a mixed‐effects framework (S‐P‐SFMM) was fit using PROC QLIM in SAS v9.4, by adding random effects to the model intercept (4).
4. *Static, individual‐level data, and linear mixed‐effects fit (S‐T‐LME):* Ducey and Knapp (2010) developed a stand density index based on tree‐level variables, which was determined to be more suitable for mixed‐species and structurally complex forests. Their approach involves fitting the following system where
  (5) $$\begin{array}{cc} \alpha_{0}x_{0}+\alpha_{1}x_{1}+\epsilon & =1,\;\text{where}\\ x_{0} & =\sum_{j}{\text{EF}}_{\mathrm{iz}}{\left(d_{\mathrm{zij}}\right)}^{1.6}\\ x_{1} & =\sum_{j}{\text{EF}}_{\mathrm{iz}}{\text{SG}}_{\mathrm{zij}}{\left(\frac{d_{\mathrm{ijz}}}{25}\right)}^{1.6} \end{array},$$
  ${\text{EF}}_{\mathrm{iz}}$ is the expansion factor for the *z*‐th tree within the *i*‐th plot; $d_{\mathrm{zij}}$ and ${\text{SG}}_{\mathrm{zij}}$ are diameter and specific gravity, respectively for the *j*‐th measurement of the *z*‐th tree at the *i*‐th plot. Ducey and Knapp (2010) did not provide an approach for estimating a self‐thinning line, but for computing the maximum carrying capacity, which can be obtained by
  (6) $$N_{\max_{i}}=\frac{100}{\alpha_{0}+\alpha_{1}\left({\bar{\text{SG}}}_{i}\right)},$$
  where $N_{\max_{i}}$ is the maximum density for the *i*‐plot and ${\bar{\text{SG}}}_{i}$ is the average‐specific gravity for that plot. Model (5) was fitted as a quantile regression model with mixed‐effects using the 95th percentile.
5. *Dynamic, site‐level data, and density model with linear mixed‐effects (D‐P‐LME)*: All the above‐explained strategies used static data, which do not require plots that have been remeasured through time. An alternative is using dynamic data, which are plots with one or more remeasurements, for deriving self‐thinning lines. Although there is imbalance between static and dynamic observations in this analysis, we do not believe this would greatly influence our general findings since we are interested in plot‐level trends and highlighting the differences across methods. Therefore, for the following strategies, we used 26 sample plots with dynamic data of stand variables through time.

Traditionally, in ecology, the rate of change in density has been studied as a density‐dependent phenomenon (Dennis & Taper, 1994; Gotelli, 2001), using the following general differential equation form:

(7) $$\frac{dN}{dt}=f\left(\theta ,N\right),$$

where f() is a function relating the rate of change to a vector of parameters θ and *N* is population density. García (2009) proposed to relate the rate of density change in terms of forest height (*H*) instead of time, and proposed the use of the following differential equation form:

(8) $$\frac{dN}{dH}=\beta_{0}N^{\beta_{1}}H^{\beta_{2}},$$

where $\beta_{0},\ldots ,\beta_{2}$ are parameters. Zeide (2010) instead used an averaged stand diameter known as the diameter of the mean basal area tree ($d_{g}$) as:

(9) $$\frac{dN}{dd_{g}}=\beta_{0}N^{\beta_{1}}{d_{g}}^{\beta_{2}}$$

A solution of equation (9) is given by Trouve et al., (2017) as the following density model:

(10) $$\ln N_{1i}=\ln\left(N_{0i}^{1-\beta_{2}}+\exp\left[\beta_{0}\right]\cdot \frac{1-\beta_{2}}{\beta_{1}+1}\cdot \left[d_{g_{0i}}^{\beta_{1}+1}-d_{g_{1i}}^{\beta_{1}+1}\right]\right)\frac{1}{1-\beta_{2}}+\epsilon_{i},$$

where $N_{1i}$ and $N_{0i}$ is tree density at the end and at the beginning of the period for the *i*‐th plot, respectively; $d_{g_{0i}}$ and $d_{g_{0i}}$ is the stand quadratic mean diameter at the end and at the beginning of the period for the *i*‐th plot, respectively. $\beta_{0},\beta_{1}$, and $\beta_{2}$ are parameters to be estimated and $\epsilon_{i}$ is a random error for the *i*‐th observation from a Gaussian distribution with mean 0 and standard deviation σ.

We fit several variants of model (10) by allowing random effects on each or all of the parameters of that model. As explained above, the plot was used as the random factor in order to take into account the hierarchical structure of the data. Given that our data have a hierarchical structure, the random effects capture variation from unmeasured variables at the plot‐level and the individual‐level in plots with repeated measures. We compared models based on the Bayesian information criterion (BIC; Schwarz, 1978).

For computing the self‐thinning estimates from the dynamic density model, we follow Trouve et al., (2017) by using the following formulas:

(11) $$\text{intercept}=\frac{\hat{\beta_{0}}}{1-\hat{\beta_{2}}}+\frac{\ln\left(\hat{\beta_{2}}-1\right)-\ln\left(\hat{\beta_{1}}+1\right)}{1-\hat{\beta_{2}}}$$

(12) $$\text{slope}=\frac{1+\hat{\beta_{1}}}{1-\hat{\beta_{2}}}$$

- *Dynamic, site‐level data, and mortality model with negative binomial mixed‐effects (D‐P‐NBME):* An alternative approach is to model how density changes based on the difference in population densities on time. We fit a negative binomial generalized linear model (NBGLM) as given by Trouve et al., (2017) in their equation (8), but allowing random effects into the intercept of the model
  (13) $$\begin{array}{cc} \Delta N_{i} & ∼\text{NB}\left(\hat{\Delta N_{i}},\theta \right)\\ \ln\left(\hat{\Delta N_{i}}\right) & =\left(\beta_{0}+u_{0i}\right)+\beta_{1}\ln\left(d_{g_{0i}}\right)+\beta_{2}\ln\left(N_{0i}\right)+\ln\Delta d_{g_{i}},\\ var\left(\hat{\Delta N_{i}}\right) & =\hat{\Delta N_{i}}+\frac{\hat{\Delta N_{i}^{2}}}{\theta }, \end{array}$$
  where: $\Delta N_{i}=N_{0i}-N_{1i}$; $\Delta d_{g_{i}}=d_{g_{1i}}-d_{g_{0i}}$; where $u_{0i}$ are plot‐specific random effects to be predicted and assumed to follow a Gaussian distribution having an expected value of 0 and variance $\sigma_{0}^{2}$; and θ is an overdispersion parameter that allows the variance to be scaled as the square of fitted $\hat{\Delta N_{i}}$ values; and the other model terms have been explained above. Note that the last term in equation (13) does not have a coefficient, which was achieved by using the option offset implemented in R as suggested by Trouve et al., (2017). In this way, the model is forced to use the measured value of $\Delta d_{g_{i}}$ as it is.

The corresponding self‐thinning line is obtained as in the previous modeling strategy, by substituting their respective parameter estimates in equations (11) and (12).

- *Dynamic, site‐level first‐measurement data and average individual size model with nonlinear mixed‐effects (D‐P‐NLME):* Hann et al., (2003) proposed a method for estimating the maximum size—density trajectory. In general, this approach differs from the others explained above in the sense that it (a) predicts stand diameter instead of tree density (like in Yoda et al., 1963), and (b) uses the first available measurement for each plot to evaluate the trajectory over time.
  (14) $$\ln d_{g_{i}}=\alpha_{0}+\alpha_{1}\ln\left(N_{i}\right)-\left[\frac{{\left(\alpha_{0}\alpha_{2}\right)}^{2}}{\alpha_{0}+\alpha_{1}\ln\left(N_{1{\text{st}}_{i}}\right)-\ln\left(d_{g_{1{\text{st}}_{i}}}\right)}\right]e^{-\alpha_{3}\left[\ln\left(N_{1{\text{st}}_{i}}\right)-\ln(N_{i})\right]}$$
  where $N_{i}$ and $d_{g_{i}}$ are the tree density and stand diameter for the *i*‐th plot, while that $N_{1{\text{st}}_{i}}$ and $d_{g_{1{\text{st}}_{i}}}$ are the same variables measured for the first time at the *i*‐th plot. The self‐thinning line is obtained by using the reversed‐Reineke's equation part of (14), and solving for *N*.

### 2.3 Comparisons

We examined two important features of the assessed modeling strategies: (1) model behavior and (2) prediction of carrying capacity.

1. *Model behavior*. In order to assess how well the self‐thinning lines depicted by each modeling strategy represent the frontier relationship of population density, we plotted them in both log and untransformed space with the observed dispersion of our data.
2. *Prediction of carrying capacity*. As a proxy for the carrying capacity, we computed the maximum stand density index (SDI_max_), that is, the maximum number of trees at a given reference average individual size (in our case a diameter) that can exist in a self‐thinning population (Husch, Miller, & Beers, 1972). As the self‐thinning model provides the maximum density for a given average plot diameter (equation (3)), the SDI_max_ is predicted by
  (15) $${\hat{\mathrm{SDI}}}_{\max_{i}}={\hat{N}}_{{\max|d_{\mathrm{base}}}_{i}}$$
  where ${\hat{N}}_{\max_{i}}$ is the predicted maximum density at a base‐average tree size $d_{\mathrm{base}}$ at the *i*‐th plot. We predicted SDI_max_ using 25.4 cm as $d_{\mathrm{base}}$, for each of the modeling strategies. We compared the predicted values of SDI_max_ among each of the strategies, by computing the multiple comparison test of Scheffé (1953).

### 2.4 Modeling carrying capacity

For the best modeling strategy, we further explored the relationship between the plot‐level ${\hat{\text{SDI}}}_{\max}$ with various forest, topographic, and bioclimatic variables. We fit several models of the form

(16) $$SDI_{max_{i}}=f\left(\theta ,X_{i}\right)+e_{i},$$

where: $SDI_{max_{i}}$ is the predicted maximum stand density index at the *i*‐plot; $X_{i}$ is the predictor variables matrix (with a first column with ones) at the *i*‐plot; f() is a linear or non‐lineal function; θ is a parameter vector (i.e., coefficients) of the model; $e_{i}$ is the random error term that follows a Gaussian distribution with zero mean and variance $\sigma_{e}^{2}$.

We assessed different predictor variables for **X** in model (16), therefore having several candidate models. Some of these variables were based on the results of Weiskittel et al., (2009). We used the following response variables for representing forest features: proportion of the primary species (in our case, the ones belonging to the *Nothofagus* genus) in density, basal area, and volume; minimum, maximum, median, standard deviation, coefficient of variation, and skewness of the diameter distribution; species richness; Shannon index; and index of species relative importance. The following topographic features were also used as predictor variables: elevation, aspect, and slope as in Stage and Salas (2007). Finally, we also tested to use the bioclimatic variables as predictor variables in model (16).

The final carrying capacity model was selected after comparing the goodness‐of‐fit of the different model formulations, prediction capabilities, and the biological behavior of the resulting model. Given that we had 19 bioclimatic variables, we first found the best single predictors of carrying capacity. We then tested each of the selected predictors with the other potential predictors.

## 3 RESULTS

**Figure 3:**
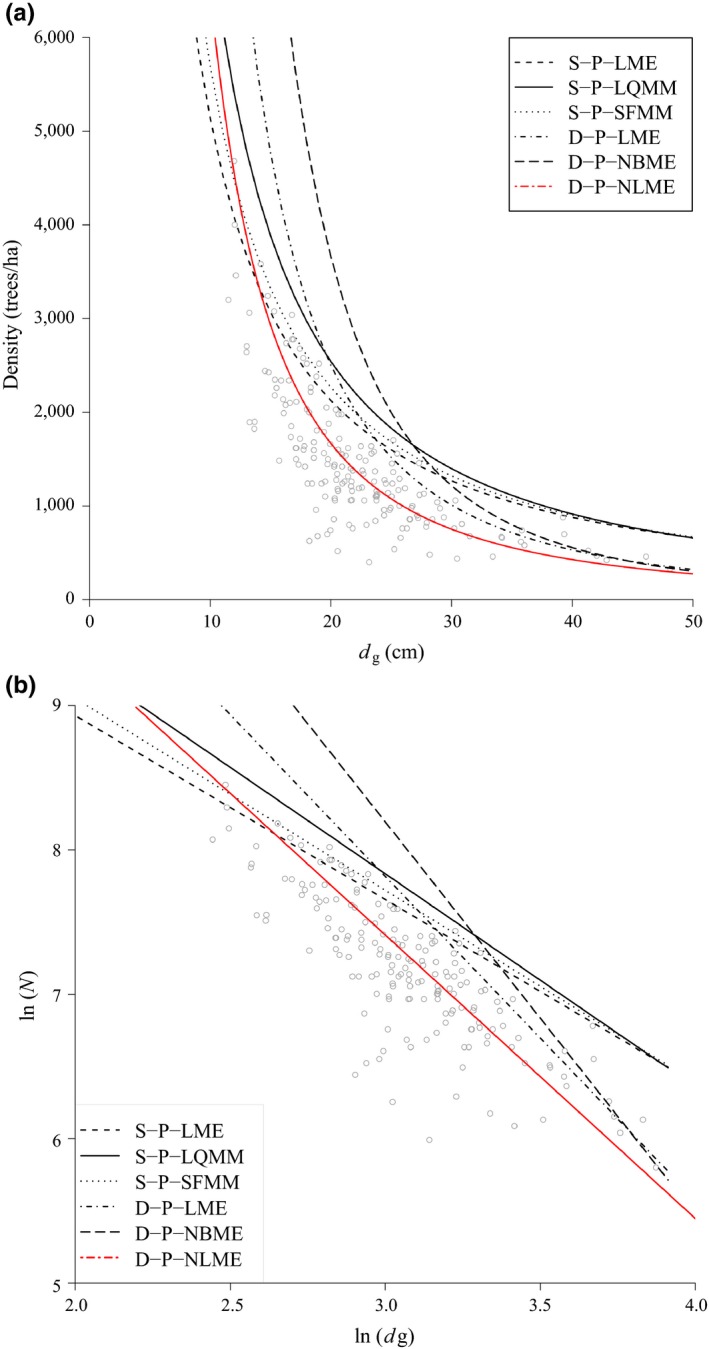
Self‐thinning lines from different modelling strategies in logarithmic scale (a) and untransformed units (b). Static site‐level mixed‐effects model (S‐P‐LME), Static site‐level quantile regression mixed‐effects model (S‐P‐LQMM), Static site‐level stochastic frontier regression mixed‐effects model (S‐P‐SFMM), Dynamic site‐level stem density mixed‐effect model (D‐P‐LME), Dynamic site‐level mortality negative binomial (D‐P‐NBME), and Dynamic site‐level based on first measurement (D‐P‐NLME) represents to model (3), (10), (13), and (14), respectively

Based upon all of the modeling strategies explained above, we predicted the self‐thinning lines both in logarithm scale (Figure 3a) and in untransformed density units (Figure 3b). The dynamic ‐ mortality (D‐P‐NBME) model strategy followed by the dynamic ‐ density model (D‐P‐LME) gave rather high self‐thinning lines. Not only is this behavior unsupported by our observed data, but also is inconsistent with the current knowledge of *Nothofagus* forest dynamics (Pollmann, 2003; Veblen, 2007; Veblen & Ashton, 1978; Veblen et al., 1979; Veblen et al., 1996; Veblen et al., 1981). The first‐measurement strategy (D‐P‐NLME) did not provide an appropriate self‐thinning line for our observed data (Figure 3b). On the contrary, both static plot‐level strategies (S‐P‐LME and S‐P‐LQMM) offered us the best behaviorof the observed data, by capturing the limiting relationship of population density as individual average size increased. Not only did the estimated intercept and slope parameters for the self‐thinning line differ among modeling strategies, but also their variances (Table 3).

**Table 3:** Estimated intercept and slope for the self‐thinning line for each modeling strategy

Modeling strategy	Estimated $\hat{\beta_{0}}$	Parameters $\hat{\beta_{1}}$
S‐P‐LME	11.4787	−1.2741
S‐P‐LQMM	12.257	−1.4742
S‐P‐SFMM	11.71	−1.33
D‐P‐LME	14.535	−2.2396
D‐P‐NBME	16.352	−2.7194
D‐P‐NLME	–	–

Note

We do not report the parameters for the D‐P‐NLME strategy, because the parameters are not directly comparable with the other strategies.

Although both S‐P‐LME and S‐P‐LQMM modeling strategies provided the closest approximation to our data, we believe S‐P‐LQMM is more suitable for self‐thinning estimation because it was able to better capture the frontier relationship of density, even if it was a bit higher than the observed maximum densities. This method could serve as a way for accounting for the sampling error scheme used for collecting the data, in the sense that we might not be able to sample some locations with higher densities. Furthermore, a quantile regression model, as our S‐P‐LQMM, makes no assumptions about the distribution of the residual error, which allows correct inference about other quantiles.

There were also important and statistically significant differences in determined carrying capacity from all of the modeling strategies. The overall mean value was 1429 ± 121 (mean ± *SE*) yet ranged from 929 to 3,900 depending on the method. Mean values by method differed by over 68%, highlighting the large differences across the modeling strategies. In general, the predicted carrying capacity was higher for the individual‐level and the dynamic strategies (Table 4). The only exception to this trend was the D‐P‐NLME strategy where the predicted carrying capacity was the lowest.

**Table 4:** Mean predicted carrying capacity for each modeling strategy

Modeling strategy	Carrying capacity (trees/ha)	Scheffe test
S‐T‐LME	1780.6	a
D‐P‐NBME	1764.2	a
D‐P‐LME	1444.2	b
S‐P‐LQMM	1349.8	b
S‐P‐SFMM	1272.2	bc
S‐P‐LME	1194.3	bc
D‐P‐NLME	1047.0	c

Note

Scheffe's multiple comparison test results at 5% significance level (different letters represent statistical differences).

The Scheffe's test delineated three distinct groups: The first one was formed by the individual‐level and the dynamic mortality model; the second one was formed by the dynamic density model, followed by static, site‐level quantile regression, stochastic regression, and linear mixed‐effects approaches; and the third one was for D‐P‐NLME. Although no statistical differences were detected, the S‐P‐LME strategy was much closer to the predictions given by D‐P‐NLME than quantile regression (S‐P‐LQMM) and stochastic regression. In addition, the average predicted SDI_max_ for the D‐P‐LME strategy was much higher than any observed forests at a given index diameter. Both S‐P‐LQMM and S‐P‐SFMM provided maximum population densities differentiating by only 78 trees/ha, but S‐P‐LQMM overall predicted value was closer to the overall mean value for a secondary *Nothofagus* forests with an average size in diameter of 25 cm. In addition, S‐P‐SFMM provided several plot‐level values that were generally too low, while S‐P‐LQMM was more consistent and had an narrower range of plot‐level values. Therefore, we favored the predictions of carrying capacity by the static, site‐level quantile regression mixed‐effects model strategy (S‐P‐LQMM).

**Figure 4:**
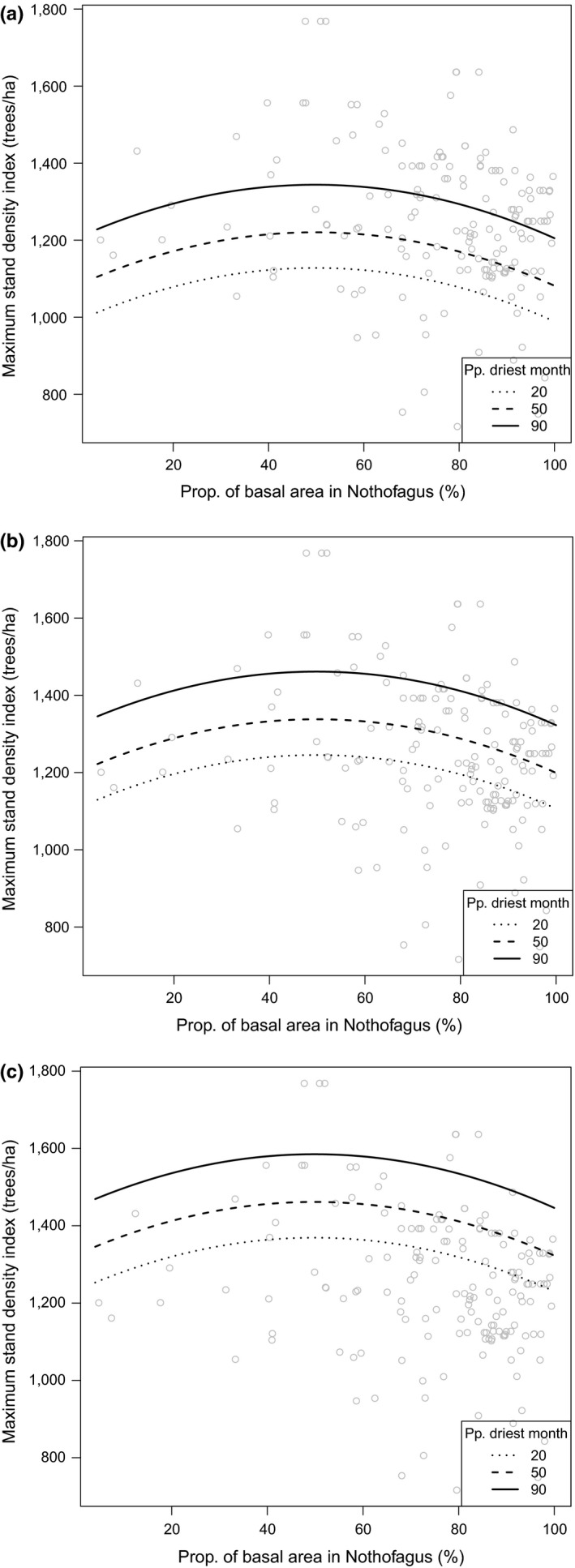
Behavior of carrying capacity depending on: dominance of pioneer species (i.e.,proportion of basal area in *Nothofagus* species); a climate variable (i.e., precipitation in the driest month); and tree species diversity (i.e., Shannon index). Carrying capacity is represented by *SDImax*. Species diversity levels were set to low (a), medium (b), and high (c) by using the values of 0.05, 1, and 2 for the Shannon index, respectively

Based on a variety of alternative models, carrying capacity was found to be effectively modeled as a function of climatic, species diversity, and abundance of pioneer species composition. The best model used variables representing: species diversity (i.e., Shannon index) and climatic conditions (i.e., precipitation in the driest month). The model had an error of about 13.5% with respect to the mean observed value of SDI_max_. Furthermore, the overall fit of the model was significantly better than all of the other models examined. The behavior of the carrying capacity model is shown in Figure 4 by using fixed (low, mean, and high) values of the predictor variables of precipitation in the driest month and Shannon's index. The results suggest that carrying capacity had a slight unimodal relationship with pioneer species composition, but was much more sensitive to precipitation in the driest month and Shannon's index, which showed positive relationships with carrying capacity.

## 4 DISCUSSION

In this analysis, the difficulty of determining site‐level carrying capacity was demonstrated given the range of contrasting values obtained from the various alternative strategies examined. To our knowledge, relatively few studies have examined the effects of contrasting modeling strategies using the same dataset, while simultaneously accounting for hierarchies in the data. Overall, we indicated that the relatively simplistic static data covering a range of conditions and a suitable statistical model, which addressed the hierarchical structure of the data, produced the most reliable results for estimating self‐thinning and carrying capacity. This is important as it allows the larger dataset to be used and suggests that remeasurements may not provide more robust estimates of self‐thinning behavior. It is important to highlight that the S‐P‐LQMM strategy provided both a suitable approach for frontier estimation and a method for assessing the site‐level factors that may influence the carrying capacity.

The high variation of the predicted carrying capacity among the various strategies indicates that the assumptions and results from any alternative must be reviewed carefully. For example, some methods had relatively high variation in estimated site‐level carrying capacity, while others showed much more limited variation. This would strongly influence ecological inference and potential additional relationships examined as was conducted in this particular analysis.

Although the individual‐level‐based strategy (S‐T‐LME) has been devised for mixed‐species forests like the ones examined in this analysis, we believe the method gave too wide of a range for carrying capacity and the overall mean that was too high. In comparison with the site‐level LQMM strategy (S‐P‐LQMM), the individual‐level‐based strategy (S‐T‐LME) provided an estimate of site‐level carrying capacity that was 32% higher. Likewise, the dynamic‐based strategies tend to provide estimates too high for the self‐thinning line. This may indicate that dynamic methods may be overly sensitive to mortality dynamics and too limited to represent a broad range of conditions given the strong reduction in available data for this method. Nonetheless, the density model (equation (10)), which was based on dynamic data, may be better suited for estimating density trajectories as in traditional growth models, such as in García (2009). However, the observed trends from the dynamic data in this analysis may be influenced by the smaller dataset used and assessment with much larger datasets should be conducted.

As found in this analysis, carrying capacity was not independent of site‐specific conditions. Prior research on self‐thinning has suggested that the limiting relationships between population density and average individual size are independent of site factors (Pretzsch & Biber, 2005; Reineke, 1933; Yoda et al., 1963). This simplification, up to some extent, broadens the use of this concept in applied forest ecology. As previously found by other studies (Andrews et al., 2018; Bi et al., 2000; Weiskittel et al., 2009; Zhang et al., 2013), we have also shown, but for the first time in *Nothofagus* forests, that carrying capacity depends on an array of factors including site productivity, species diversity, and successional stage of development. Although several formulations could be used for representing site productivity (Stage & Salas, 2007), we focused on identifying bioclimatic variables that had a biologically consistent behavior. For this analysis, we found that the precipitation in the driest month negatively affected carrying capacity and effectively represented site productivity. Higher precipitation in the driest month resulted in a greater carrying capacity, which is consistent with biological expectations. Therefore, under the current climate change global scenario, we may expect a decline in maximum carrying capacity for the majority of this forest type, which were similar to the general recent findings for Andrews et al., (2018) for the Acadian Region of North America.

Interestingly enough, species diversity (represented by the Shannon index) may help to overcome the effect of adverse climatic conditions as greater species diversity increased site‐level carrying capacity. We think that is because of the resource‐use differentiation among different functional groups. In addition, the proportion of basal area in the pioneer species (a proxy for stage of development) has a quadratic effect into carrying capacity. Our results indicated that there was an optimum proportion of basal area occupied by pioneer species, which was approximately 50% in this analysis. Therefore, pure *Nothofagus* forests will not likely achieve the maximum potential carrying capacity. This finding is in line with the additive basal area concept (Lawes et al., 2006; Lusk, 2002), which has also been suggested in *Nothofagus* forests as well (Donoso & Lusk, 2007; Donoso & Soto, 2016). This idea suggested that both shade‐tolerant species contributed in adding more basal area to a forest because of resource‐use differentiation among various functional groups. Our findings are also in agreement with current research on assessing the mixing of species in tree growth (Piotto, 2008) as well as forest productivity worldwide (Liang et al., 2016). However, our findings differ from those of Weiskittel et al., (2009) who found that site‐level carrying capacity increased with primary species composition purity for three species in the Pacific Northwest, USA. This may highlight an important distinction between temperate plantations and natural rainforests as were examined in this particular analysis.

## 5 CONCLUDING REMARKS

The modeling strategy involving the use of static, population‐level data and linear quantile regression mixed‐effects provided a reliable ecological behavior for both self‐thinning estimation and modeling carrying capacity. The type of data (i.e., static and dynamic) heavily influenced the findings for self‐thinning and carrying capacity with dynamic methods tending to provide much higher estimates. In particular, the density model based on dynamic data tended to overestimate the self‐thinning line, but could likely be a suitable tool for growth modeling. By fitting the equations in a mixed‐effects framework, the evaluation of various external factors that may influence carrying capacity could be assessed. In this analysis, climatic, stage of development, and species diversity were found to be influential. Although the analysis highlighted the strong influence of modeling strategy on self‐thinning and maximum carrying capacity, the data, particularly the dynamic data, were relatively small despite covering a wide range of conditions. Additional analyses using more extensive datasets across a variety of species are likely necessary to verify the findings presented for this analysis. Overall, the findings highlight the challenge in identifying and defining self‐thinning relationships and maximum carrying capacity despite being fundamental concepts in applied ecology and management.

## AUTHOR CONTRIBUTION

The study was designed by CS and ARW, and CS collected the data with help from different sources. CS analyzed the data and wrote the manuscript, with input from ARW.
